# Supplementary material for: Lockdown’s Silver Lining? Different Levels of Roadkill during the COVID-19 Times in Lithuania
Source: Animals (Basel). 2023 Sep 14;13(18):2918. doi: 10.3390/ani13182918 (PMC10525093; doi:10.3390/ani13182918)
Supplement: Supplementary file 1 [file animals-13-02918-s001.zip › animals-2592381-supplementary.pdf]

Supplementary materials

Table S1. Mammal and roe deer roadkill sample sizes on main, national and regional roads, 2002–2022, and other roads, 2007–2022. MVC: mammalian roadkills, RDVC: roe deer roadkills.

Year	Main roads			National roads			Regional roads			Other roads		
	**MVC, N**	**RDVC, n**	**RDVC, %**	**MVC, N**	**RDVC, n**	**RDVC, %**	**MVC, N**	**RDVC, n**	**RDVC, %**	**MVC, N**	**RDVC, n**	**RDVC, %**
2002	126	42	33.3	188	93	49.5	59	15	25.4			
2003	171	52	30.4	207	92	44.4	69	23	33.3			
2004	240	104	43.3	243	128	52.7	88	33	37.5			
2005	243	102	42.0	306	152	49.7	92	41	44.6			
2006	271	105	38.7	381	201	52.8	129	49	38.0			
2007	658	158	24.0	632	257	40.7	171	79	46.2	164	41	25.0
2008	658	145	22.0	588	258	43.9	169	84	49.7	119	36	30.3
2009	1980	159	8.0	507	255	50.3	148	73	49.3	139	44	31.7
2010	413	135	32.7	548	308	56.2	192	95	49.5	197	69	35.0
2011	402	129	32.1	487	244	50.1	176	94	53.4	200	78	39.0
2012	466	175	37.6	542	326	60.1	158	87	55.1	213	83	39.0
2013	775	180	23.2	850	408	48.0	250	126	50.4	245	111	45.3
2014	1131	283	25.0	995	525	52.8	385	229	59.5	299	161	53.8
2015	862	336	39.0	1156	679	58.7	458	295	64.4	348	176	50.6
2016	863	403	46.7	1454	988	68.0	573	402	70.2	437	282	64.5
2017	684	341	49.9	1058	735	69.5	419	294	70.2	393	223	56.7
2018	953	548	57.5	1700	1283	75.5	747	576	77.1	665	463	69.6
2019	1154	733	63.5	2003	1495	74.6	895	724	80.9	975	700	71.8
2020	924	505	54.7	1516	1060	69.9	658	503	76.4	1619	1325	81.8
2021	921	513	55.7	1837	1357	73.9	904	696	77.0	1619	1281	79.1
2022	892	573	64.2	1643	1255	76.4	807	615	76.2	1560	1230	78.8

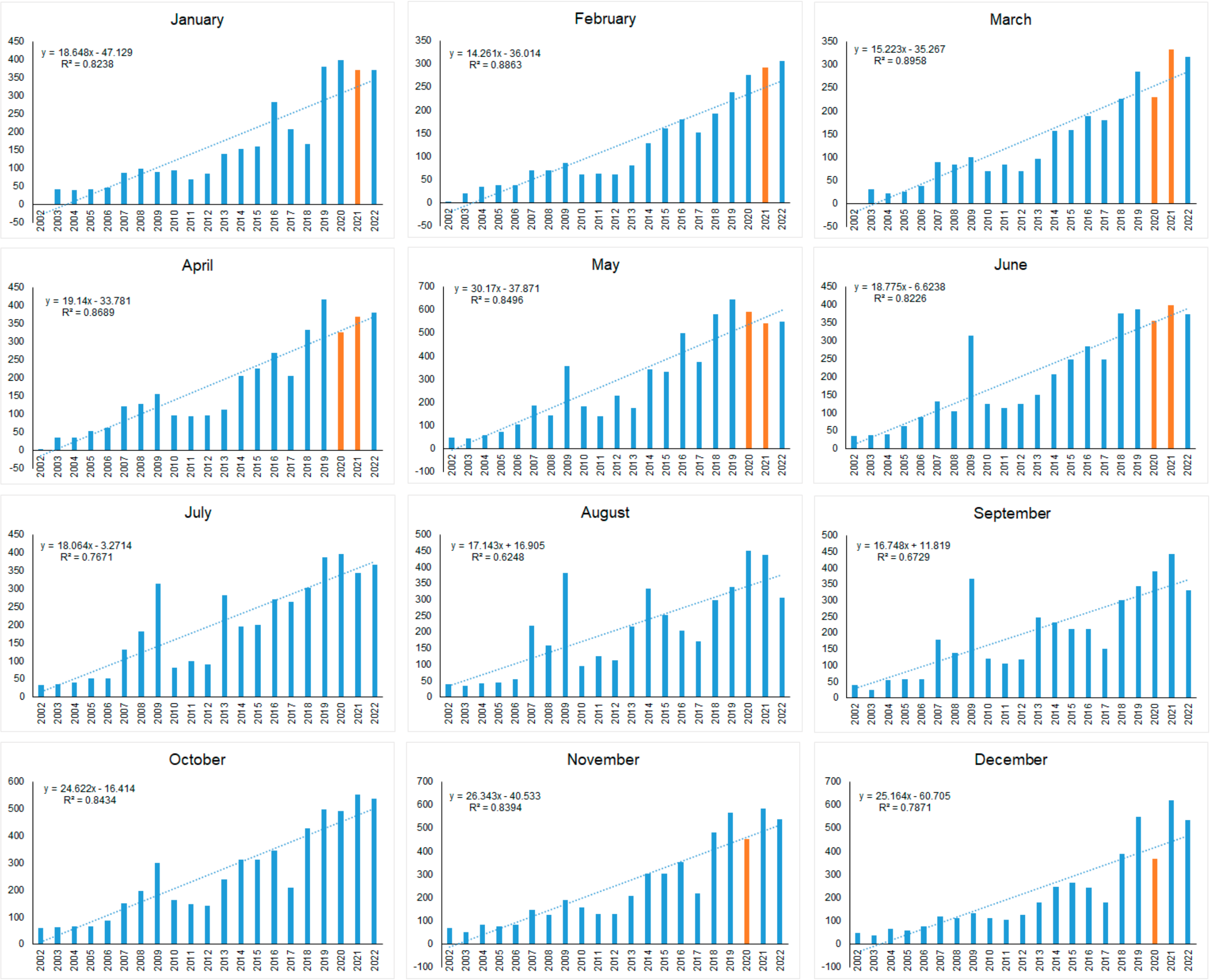

Figure S1. The monthly dynamics of MVCs on the main, national and regional roads in Lithuania from 2002 to 2022. The regression lines depict the anticipated annual roadkill figures.

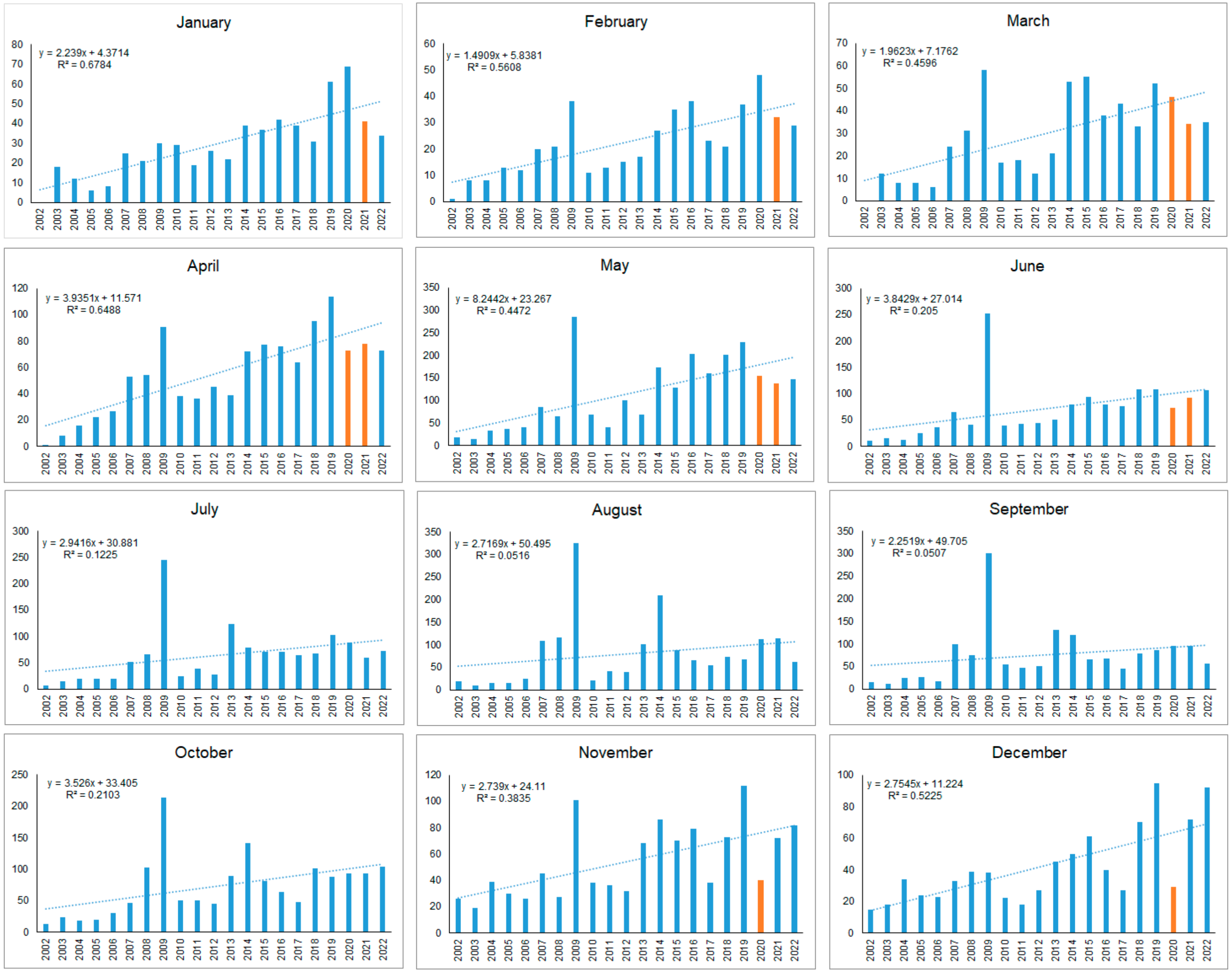

Figure S2. The monthly dynamics of MVCs on the main roads in Lithuania from 2002 to 2022. The regression lines depict the anticipated annual roadkill figures.

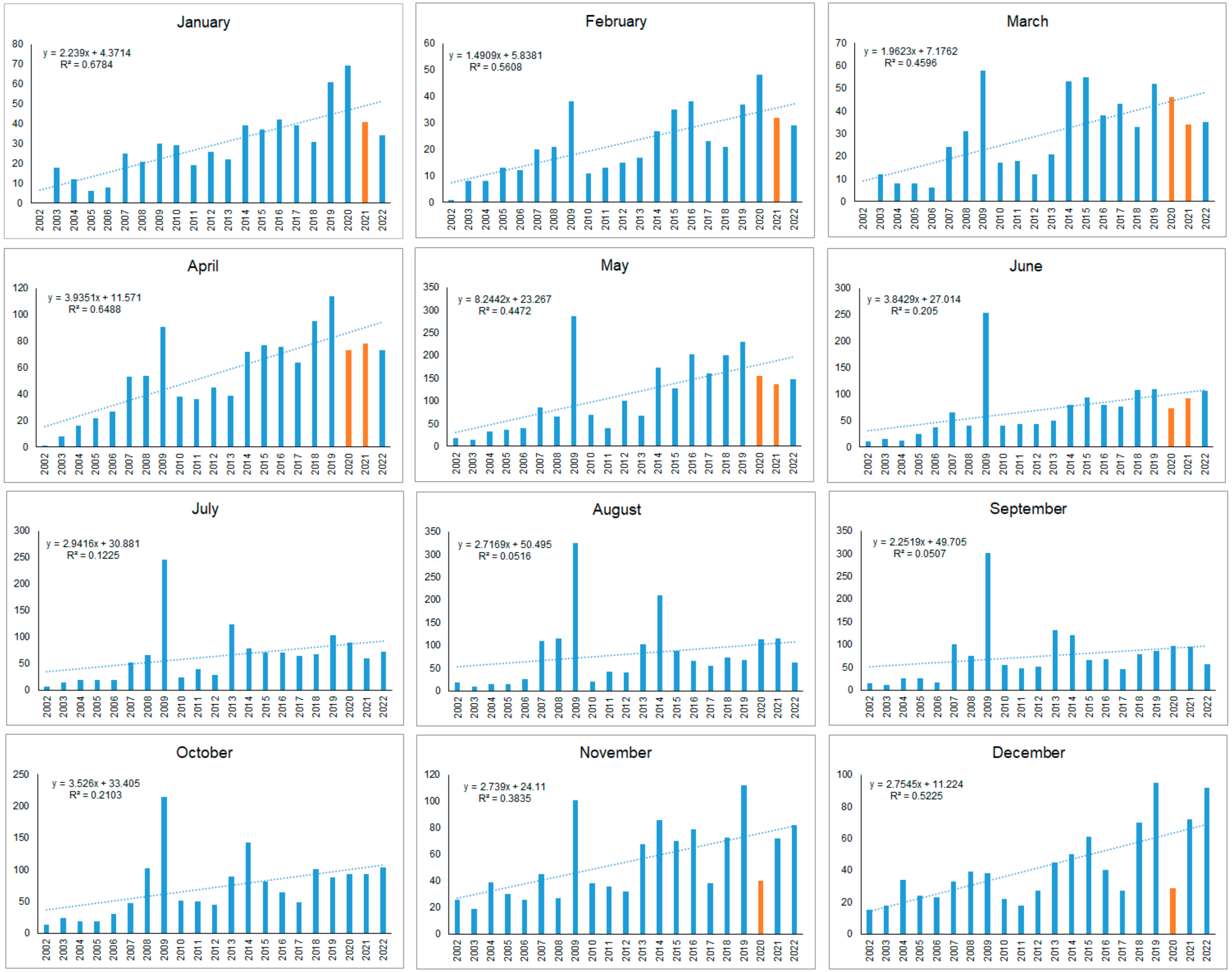

Figure S3. The monthly dynamics of MVCs on the national roads in Lithuania from 2002 to 2022. The regression lines depict the anticipated annual roadkill figures.

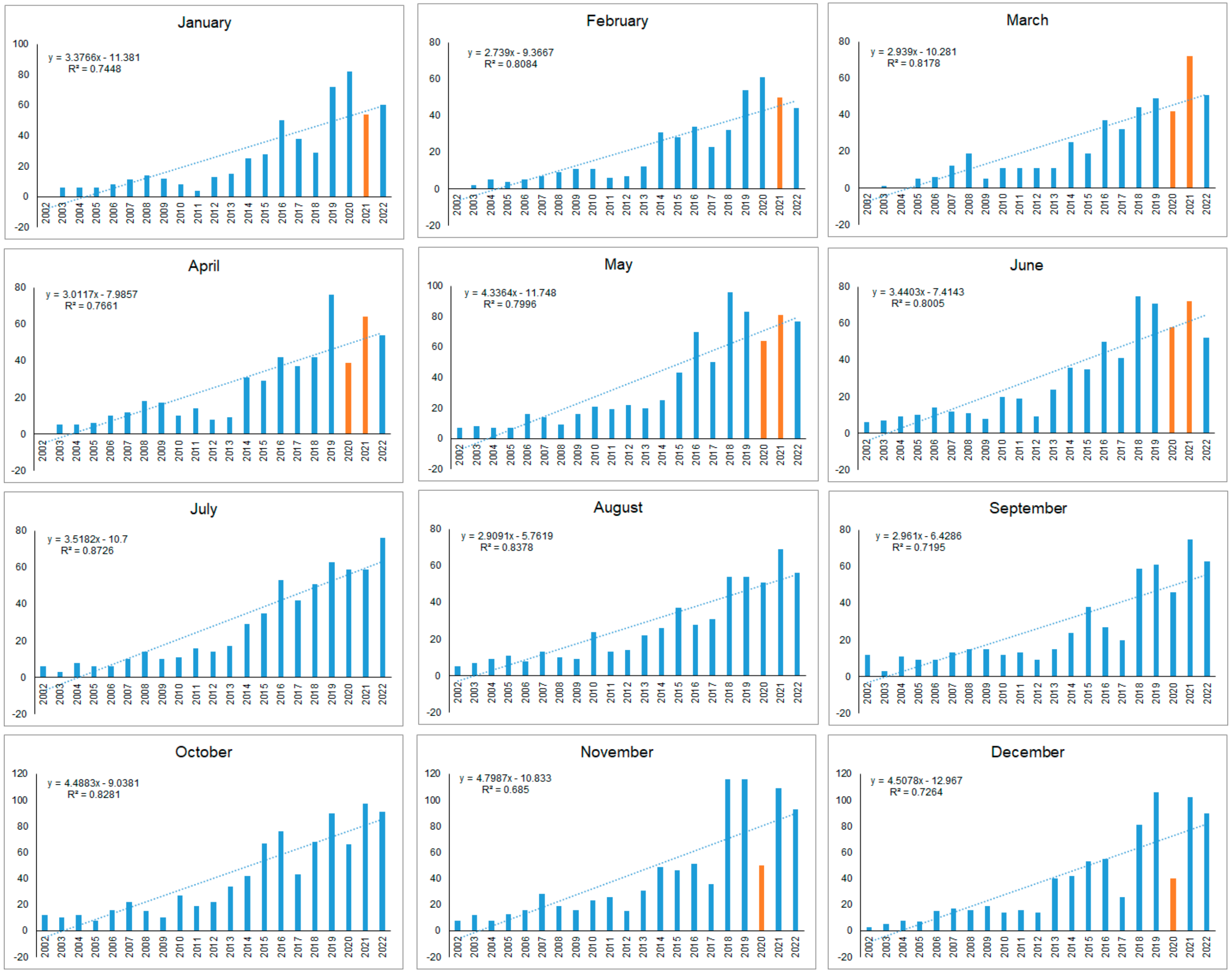

Figure S4. The monthly dynamics of MVCs on the regional roads in Lithuania from 2002 to 2022. The regression lines depict the anticipated annual roadkill figures.

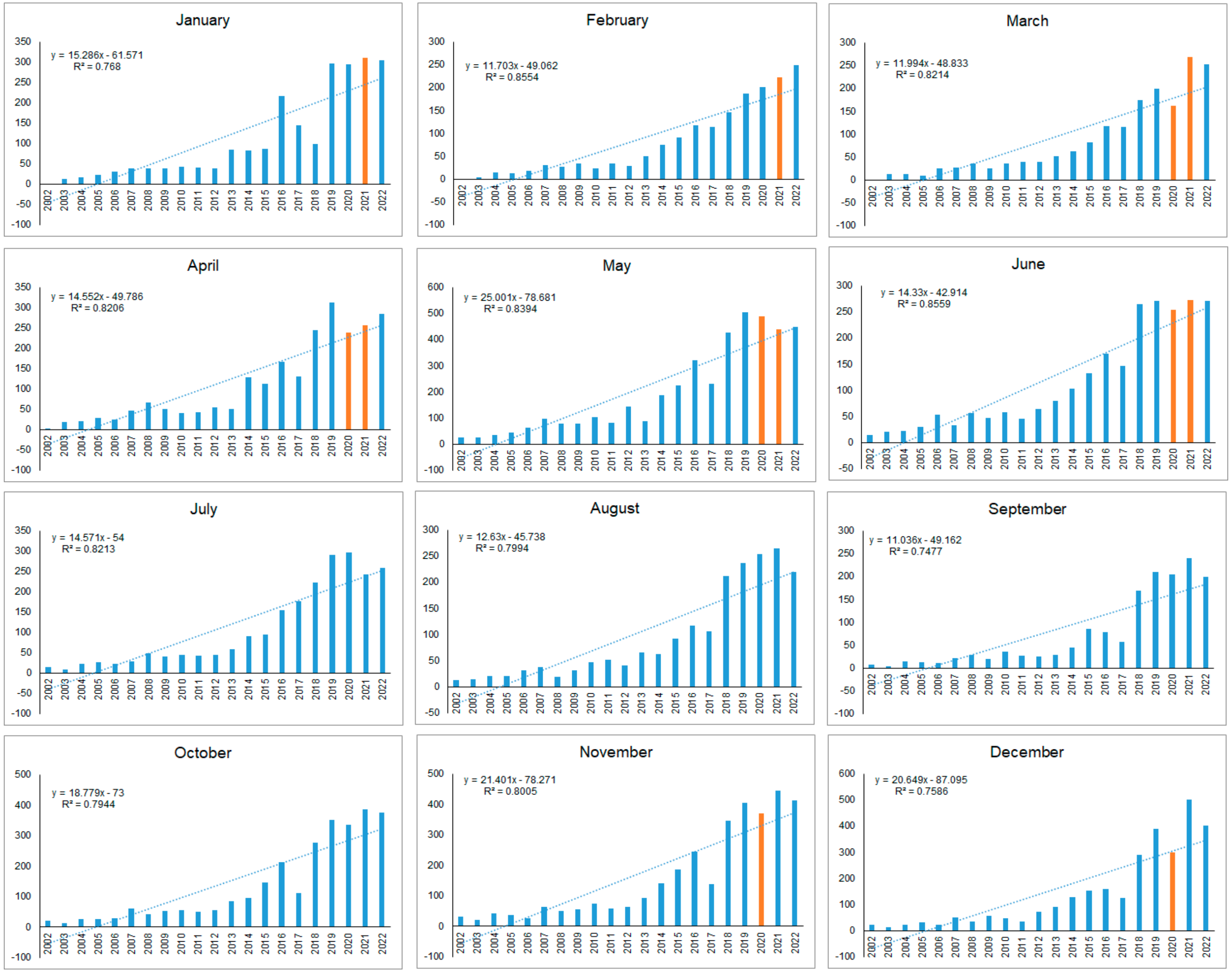

Figure S5. The monthly dynamics of RDVCs on the main, national and regional roads in Lithuania from 2002 to 2022. The regression lines depict the anticipated annual roadkill figures.

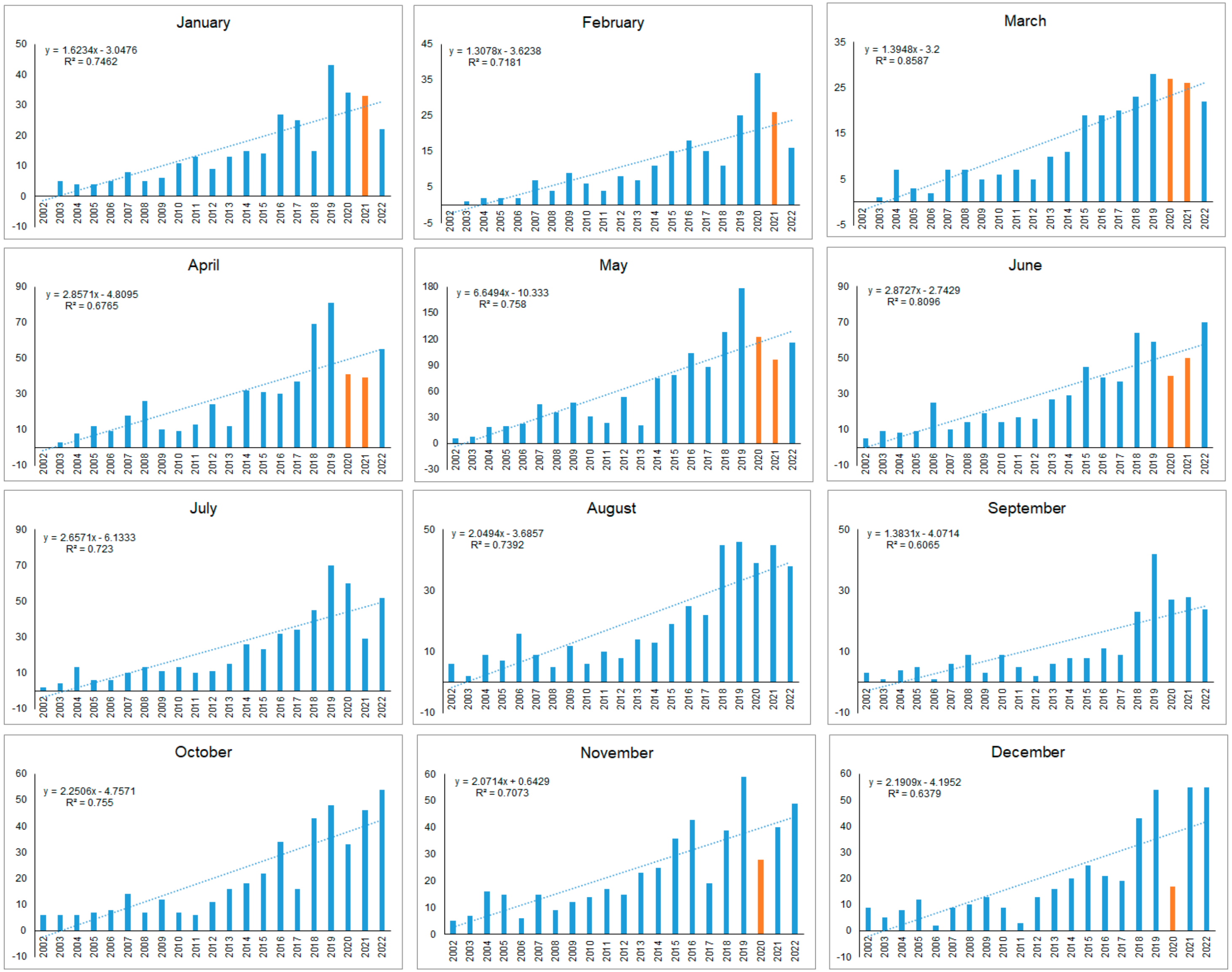

Figure S6. The monthly dynamics of RDVCs on the main roads in Lithuania from 2002 to 2022. The regression lines depict the anticipated annual roadkill figures.

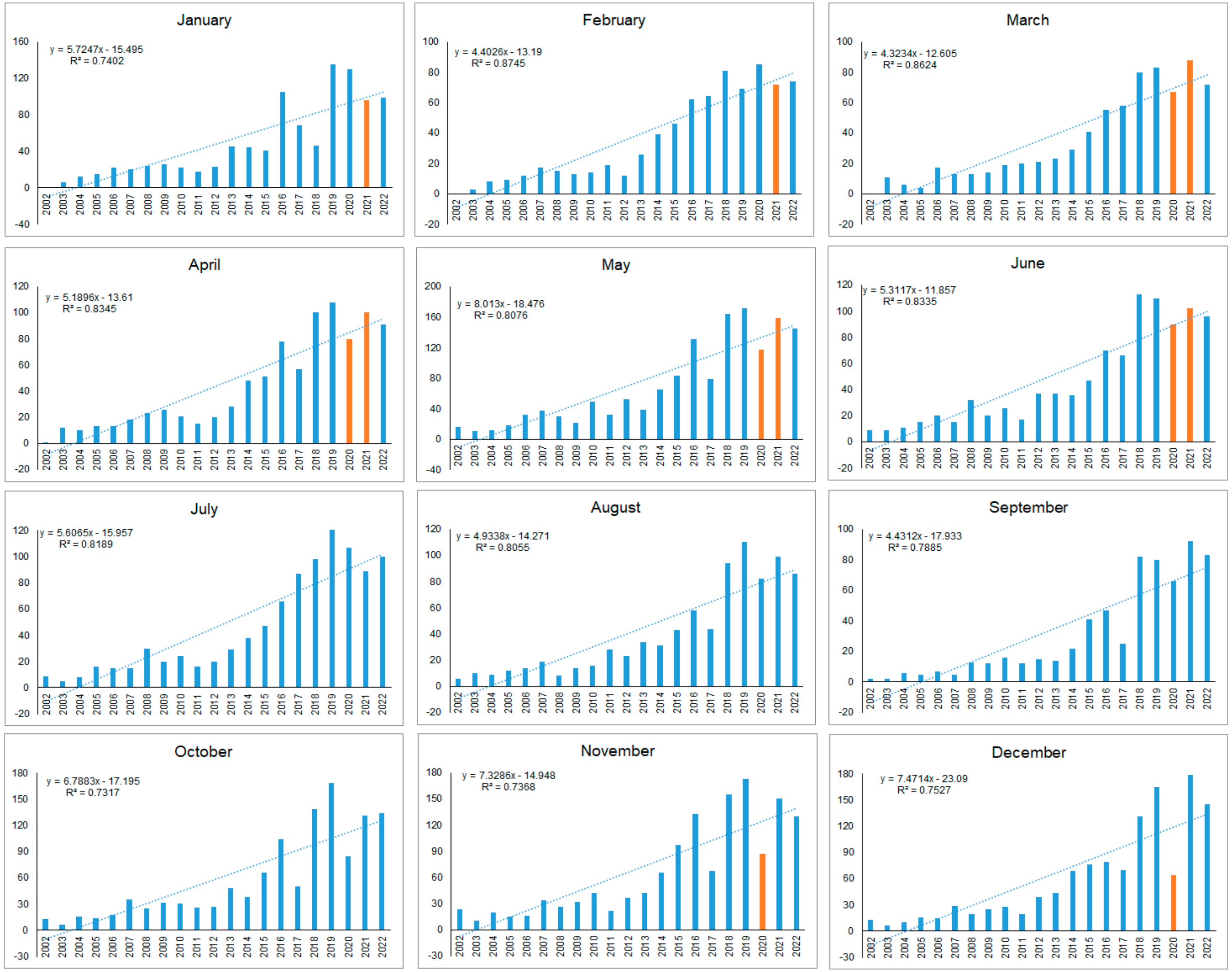

Figure S7. The monthly dynamics of RDVCs on the national roads in Lithuania from 2002 to 2022. The regression lines depict the anticipated annual roadkill figures.

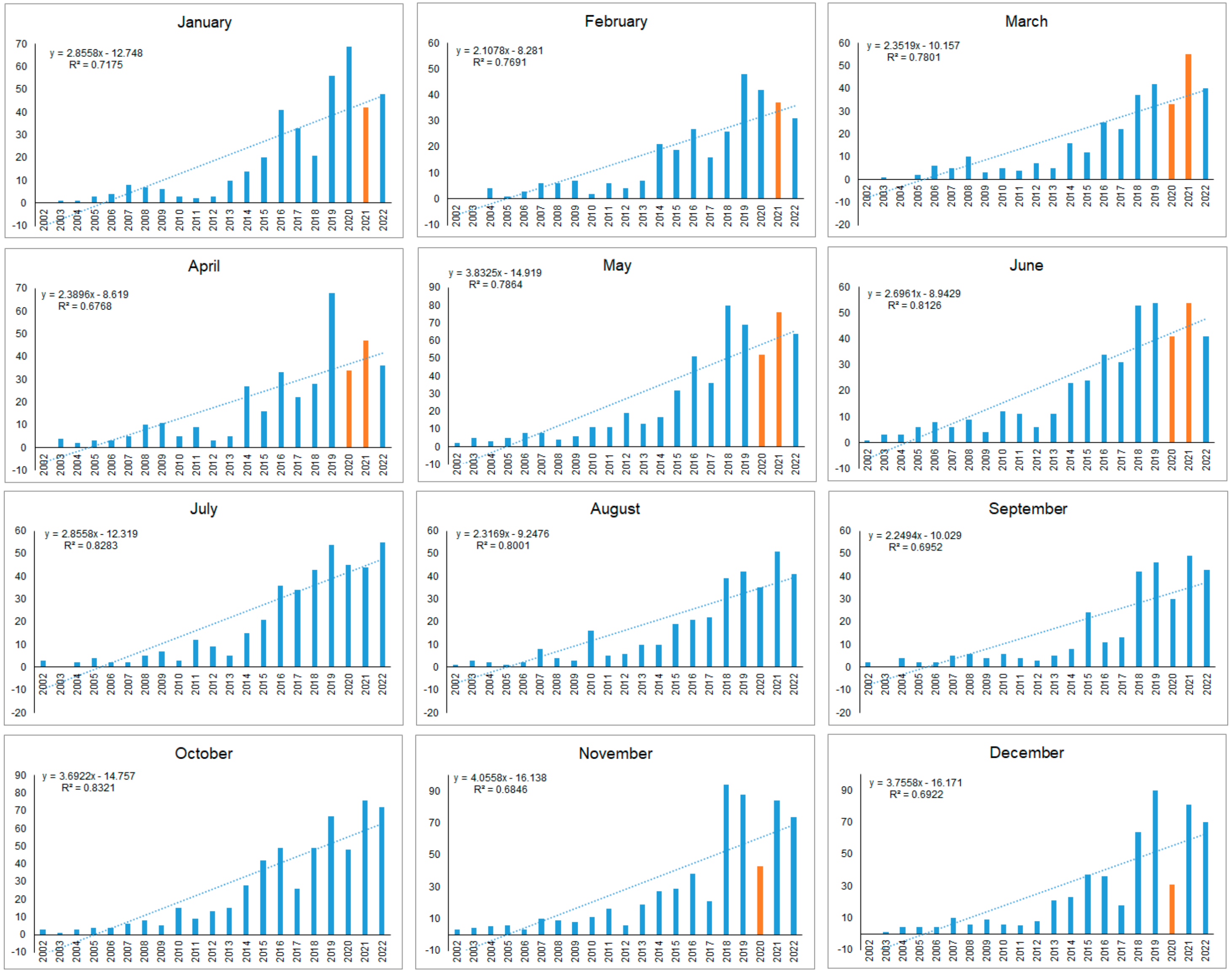

Figure S8. The monthly dynamics of RDVCs on the regional roads in Lithuania from 2002 to 2022. The regression lines depict the anticipated annual roadkill figures.
